# Cultural adaptation of an integrated eating disorders prevention and healthy weight management program

**DOI:** 10.1186/s40337-023-00950-5

**Published:** 2023-12-20

**Authors:** Courtney C. Simpson, Rachel L. Boutté, C. Blair Burnette, Madison Weinstock, Neha Goel, Suzanne E. Mazzeo

**Affiliations:** 1 Department of Psychiatry, University of California, San Diego, CA USA; 2 Department of Family and Preventive Medicine, Rush Medical College, Chicago, IL USA; 3 Department of Psychology, Michigan State University, Lansing, MI USA; 4 Department of Psychology, Virginia Commonwealth University, PO Box 842018, Richmond, VA 23284-2018 USA

**Keywords:** Eating disorders, Body image, Prevention, Appearance ideals, Black/African Americans, Latinas

## Abstract

**Background:**

Both eating disorder (ED) prevention and weight management interventions often focus on the thin ideal. Yet, many Black and Latina women do not view thinness as their body ideal. This study used focus groups to investigate the influence of race, ethnicity, and culture on appearance ideals and inform the cultural adaptation and integration of two established programs addressing EDs and weight management: the Body Project, and Healthy Weight Interventions.

**Methods:**

White (*n* = 10), Black (*n* = 14), and Latina (*n* = 6) women participated in racially and ethnically homogenous focus groups.

**Results:**

Thematic analysis identified several themes, including: (1) diverse beauty standards across groups, (2) lack of acknowledgement of racialized beauty standards in prevention and cultural appropriation, (3) culturally-specific impacts of standards, (4) harm of appearance-related comments, (5) limitations of available resources, (6) stigma/minimization of mental health, (7) barriers to inclusive programming, and (8) facilitators of inclusive programming.

**Conclusions:**

Results suggested that current programs’ emphasis on thinness limit their relevance for women of color, and perpetuate the misconception that EDs primarily affect White women. Findings highlight the need for culturally responsive prevention.

## Abstract (plain language summary)

Both eating disorder (ED) prevention and weight management interventions often focus on thinness as the ideal. Yet, many Black and Latina women do not view thinness as their ideal body shape. This study interviewed women in focus groups to investigate the influence of race, ethnicity, and culture on appearance ideals and inform the cultural adaptation and integration of two established programs addressing EDs and weight management: the Body Project, and Healthy Weight Interventions. White (*n* = 10), Black (*n* = 14), and Latina (*n* = 6) women participated in racially and ethnically homogenous focus groups. Several themes emerged, including: (1) diverse beauty standards across groups, (2) lack of acknowledgement of racialized beauty standards in prevention and cultural appropriation, (3) culturally-specific impacts of standards, (4) harm of appearance-related comments, (5) limitations of available resources, (6) stigma/minimization of mental health, (7) barriers to inclusive programming, and (8) facilitators of inclusive programming. Results suggested that current interventions over-emphasize thinness, limiting their relevance for women of color, and perpetuate the misconception that EDs primarily affect White women. Findings highlight the need for more culturally responsive approaches to prevention.

## Introduction

Both eating disorders (EDs) and high body weight are associated with significant morbidity and mortality [20, 28]. These conditions affect women across ethnic and racial groups [25], and are often chronic [33], highlighting the importance of prevention. The strong association between ED symptomatology and high body weight has long been noted [32], and, although many individuals with high body weight do not engage in unhealthy eating behaviors, these conditions often co-occur [60], supporting an integrated prevention approach, particularly in higher-risk groups, such as emerging adult women [25].

Indeed, college women are an important focus of prevention efforts addressing both EDs and healthy weight management. EDs and related symptomatology are not uncommon in this demographic group [25]. Moreover, diet quality and physical activity decline throughout college, a developmental stage in which long-term health-related behaviors are established [9]. Nonetheless, few prevention efforts focus on emerging adults, and most target either EDs or weight management [38]. Moreover, their relevance for women of color is limited [41].

### ED prevention and healthy weight management

Within the area of ED prevention, dissonance-based interventions (DBIs) are commonly implemented and researched. DBIs are predicated on Festinger’s Cognitive Dissonance Theory, which posits that psychological tension arises when people’s behaviors are inconsistent with their beliefs and/or attitudes [17]. Thus, DBIs aim to shape attitudes and beliefs related to behaviors that individuals are attempting to change. ED-related DBIs including Stice’s Body Project, primarily target thin-ideal internalization by changing participants’ beliefs and attitudes about the thin ideal, with the ultimate goal of preventing disordered eating behaviors. Indeed, these treatments have been shown not only to reduce thin ideal internalization but also have repeatedly yielded significant effects on multiple ED outcomes (e.g., body dissatisfaction, dieting, future ED onset; [2]. The Healthy Weight Intervention (HWI) initially was designed by Stice and colleagues to serve as a placebo control group in a trial evaluating The Body Project [48]. The HWI encourages participants to pursue the, “healthy ideal” through gradual diet and exercise changes [52].

Although the HWI was designed as a control intervention, results of an initial trial indicated that participants in this group manifested reductions in bulimic and dieting behaviors, body dissatisfaction, and negative affect [48]. Long-term follow-up supported these findings [54]. Nonetheless, concerns about the cultural relevance of both the Body Project and the HWI remain.

### The Body Project’s cultural relevance

The Body Project’s emphasis on thin-ideal internalization might not be universally appropriate, as both Black and Latina women have reported preferring curvaceous body shapes [1, 43]. For example, recent research has identified an increased pressure to obtain an hourglass or curvy body shape, characterized by a wider, curvier chest and hips, and a thin waist [19, 22]. Black and Latina women appear to endorse curvy ideals more than White women [34, 45]. Thus, measures and interventions centered on thinness likely overlook the primary body image concerns of women from these groups.

Despite differing beauty ideals, the overall prevalence of EDs is comparable among White, Black, and Latina women [6, 25]. Nonetheless, some group differences are evident when examining the prevalence of specific types of disordered eating behaviors and aspects of eating disorder psychopathology [42]. For example, Black women have higher rates of binge eating than White women [12], and bulimia nervosa is more prevalent among Black and Latina women than White women [29]. Weight-related attitudes and body image appear to differ across racial groups as well. Current understanding of body dissatisfaction within Black and Latina groups is unfortunately limited by extant body image measures that generally focus on thinness [34]. Despite evidence that disordered eating and related symptomatology appears across racial and ethnic groups, The Body Project, like other ED prevention programs, was initially designed and validated with White women, limiting its generalizability.

Several efforts have evaluated the Body Project’s effectiveness for ethnically and racially diverse individuals [4, 27]. Although initial investigations with diverse samples yielded promising results, these studies generally included small samples, and selection biases and differing risk factors might have influenced outcomes [40, 50]. Moreover, peer leaders implementing the Body Project recommended this intervention incorporate a greater emphasis on body ideals of racially and ethnically diverse individuals [59]. Culturally sensitive ED prevention that considers broader beauty ideals could improve program acceptability and reduce attrition, which is particularly important for emerging adults from marginalized racial and ethnic groups who are less likely to receive ED treatment [30].

### Cultural relevance of healthy weight management programming

Healthy weight management programs also lack cultural relevance for many women of color. Black and Latina women are more likely than their White peers to have both high body weights [44], and associated comorbidities, including diabetes [37]. Yet, only a few programs have intentionally used culturally sensitive weight management approaches for Black or Latina women, and these have typically enrolled older individuals [37]. Cultural adaptations of these programs have typically used multiple strategies, including translating materials into Spanish, emphasizing the role of social support systems, integrating relevant cultural beliefs and traditions, and including recipes that are culturally relevant [24, 37]. Nonetheless, some have suggested that weight management efforts further marginalize already minoritized groups by centering weight loss as a primary outcome, implying that the ideal body is a thin one [13]. There remains an urgent need to enhance understanding of these groups’ perspectives regarding optimal eating and activity related behaviors [37]. This work also needs to include younger women, and incorporate culturally sensitive strategies to ameliorate body dissatisfaction, which is evident in Black and Latina women, but not typically addressed in weight management programming [14].

### Program tailoring

One way to enhance programming’s relevance for specific groups is to collect qualitative data addressing the needs and experiences of the community of interest; this approach is considered particularly valuable when refining interventions [8]. Focus groups are an especially beneficial way to collect qualitative data, because group dynamics can highlight subcultural values [23], guiding the creation of culturally-relevant materials [21]. In this study, we present focus group data that informed adaptations of a program integrating the Body Project and HWI approaches for racially and ethnically diverse college women at risk of developing EDs and high body weight [46].

### Summary

Given the need for research addressing the perceived relevance of ED prevention and healthy weight management programs for Black and Latina women, we conducted focus groups to: (1) enhance understanding of the influence of race and ethnicity on appearance standards and weight-related behaviors in Black, Latina, and White women, and (2) explore how two existing programs might be adapted to enhance their applicability to Black and Latina women. The goal was to integrate and expand the Body Project and the HWI, programs which have yielded promising results in emerging adult (primarily White) women, and develop an acceptable approach to the prevention of EDs and unhealthy weight management. 

## Method

### Participants

Participants (*n* = 30) attended a public university in the Southeastern United States. The university is a large, urban institution with 44.6% White students, 17.9% Black students, and 9.4% Latine students. Participants were recruited from the psychology pool and via flyers describing an opportunity to participate in a group exploring culture, body ideals, and living a healthy college lifestyle. Interested individuals completed a demographic survey and ED diagnostic screener (Eating Disorder Diagnostic Screener, EDDS; [57] via REDCap [18]. Eligible women self-identified as Black (*n* = 14), Latina (*n* = 6), or White (*n* = 10), ages 18–25, and were enrolled in college. Individuals were excluded and provided referrals if they met clinical ED criteria (measured by the EDDS), as this study addressed prevention. Additionally, individuals were excluded if their body mass index (BMI) was ≥ 30 kg/m^2^, as the of aim this investigation was focused on informing prevention programming. Participants’ mean age was 19.33 years (*SD* = 1.21); mean BMI = 23.25 kg/m^2^ (*SD* = 3.09); 56.7% were first-year students, 26.7% sophomores, 6.7% juniors, and 10.0% seniors.

### Procedure

The university’s Institutional Review Board approved this study. Participants consented prior to the online survey, and at the start of focus groups. Seven two-hour semi-structured focus groups with participants from the same racial and/or ethnic group were conducted (*n* = 2 with White women; *n* = 3 with Black women; *n* = 2 with Latina women). Groups were led by a trained doctoral student and a process observer. Leaders and process observers were members of the same racial and/or ethnic group as participants. The decision to have racially and ethnically homogenous group leaders and members was guided by research indicating that, compared with racially heterogeneous focus groups, homogenous groups, “appeared more comfortable discussing ethnic and cultural differences” [16], p. 10). A semi-structured interview guide facilitated group conversation (Appendix A). The interview guide was developed by a psychologist (SEM) and advanced psychology trainees (CSC, RLB, CBB, and NG) with expertise in the etiology, prevention, and treatment of eating disorders, body image concerns, and healthy weight management. This team reached consensus regarding question content. The interview included a discussion of appearance norms each group perceived as relevant, an exploration of factors impacting body image and eating behavior, and a review of exercises included in the Body Project and HWI. An audit trail documented procedures, discussions, and decisions, to enhance data credibility [7]. Focus groups were audio-recorded and transcribed.

### Measures

Measures were completed via REDCap [18] prior to focus groups.

***Demographics.*** Participants reported age, year in school, sex, race, ethnicity, height, and weight.

***Eating Disorder Diagnostic Screener (EDDS).*** The EDDS [57] is a self-report measure assessing *DSM-IV* ED diagnostic criteria. It yields internally consistent scores, and demonstrates convergent validity with other ED measures [49, 57].

### Data analysis

Group facilitators verified transcripts’ accuracy. Thematic analysis identified patterns and themes within transcripts [5]. An inductive coding approach, in which the data drove the conclusions rather than predetermined theory, was used to ensure results were informed by participants’ comments [10]. The research team included a Black woman and three White women. All had prior experience with qualitative ED and weight management research.

Team members independently read assigned transcripts and then met to create preliminary codes. After this meeting, the first author established a codebook and coded one transcript from each ethnic or racial group. The team reviewed these coded transcripts, and then met again to discuss codes. After refining the codebook, the first author revised the coding of the initial three transcripts and coded the remaining four using the updated codebook. The team reviewed these transcripts, made notes regarding themes, and met again to refine the codebook further. The first author then recoded all seven transcripts to maintain consistency with the final codebook. Upon coding completion, the first author grouped codes into themes [5]. The team refined themes to ensure internal and external homogeneity [36]. Discrepancies were minimal and consensus was met.

## Results

Key themes included: (1) diverse beauty standards across groups, (2) lack of acknowledgement of racialized beauty standards and cultural appropriation, (3) culturally-specific impacts of standards, (4) harm of appearance-related comments, (5) resource limitations, (6) mental health stigma/minimization, (7) inclusive programming barriers, and (8) inclusive programming facilitators. Themes are described below. Quotations include a prefix of W (White), B (Black), or L (Latina) indicating race/ethnicity, group number, and letter delineating participant.

### Diverse beauty standards

Groups began by exploring beauty ideals specific to participants’ racial and ethnic backgrounds, and perceived differences among groups. White women overwhelmingly described thinness, exemplified by a small frame, thin waist, and overall slender shape, as the ideal within their race. White participants highlighted a, “flat stomach,” [W1a], “prominent collarbones,” [W1c], and a “thigh gap,” [W1b], as important. White women also expressed awareness of differences between their racial group's beauty standards and those of others, “If you are White, then like, you have to be more [sic] thinner,” [W1e]. Some White women mentioned that standards are, “expanding a little bit…” [W2b]; White women noted that curves are coveted within the context of thinness, stating, “You have to be thin, but have curves” [W1b].

In all Black and Latina groups, negative connotations of thinness were noted. “Being skinny…like, that’s a bad thing” [B3b]. Similarly, multiple Latina participants described thinness as undesirable. “In my family and culture, if you’re too skinny, it’s not okay…[then]…they’re like,…’you’re sick’” [L2b]. Many Black and Latina women identified the, “curvy ideal” as the beauty standard within their culture.

Furthermore, women of color repeatedly highlighted the importance of non-weight related aspects of appearance. Latina women described feeling pressure to, “make sure you look presentable every day” [L1c], and to have tan, clear skin and long, dark hair. Black women similarly noted that skin tone and hair are important elements of beauty within their race. Participants described, “having your hair put together” as essential to attractiveness. Overall, Black and Latina participants highlighted the need to incorporate non-weight related appearance pressures into prevention.

### Racialized beauty standards and cultural appropriation

Women of color expressed a desire for programs to acknowledge systemic racism’s influence on appearance standards. Black women noted that, within their culture, lighter skin is considered more attractive than darker skin. Participants expressed frustration with this standard and referred to it as, “racism in our own culture” [B3a]. Multiple women also described experiencing appearance-related racial microaggressions. For example, one Black woman reported hearing others describe someone as, “pretty for a dark-skinned girl” [B4c]. Participants also highlighted the impact of cultural appropriation. They noted that Afrocentric features (e.g., full lips) are considered beautiful on Whites, but devalued among Black individuals. “Since it is common on us, it’s like they don’t care anymore” [B3f].

Women across groups also noted that it remains important to retain some content highlighting society’s idealization of thinness. Indeed, women of color described the importance of acknowledging the effects of dual appearance pressures on body image. For example, Latina women noted feeling torn between White and Latina appearance ideals, and described their body image as complicated, because they were, “not looking like [their] sorority sisters” [L1a].

### Culturally-specific impacts

Black and Latina participants discussed challenges of meeting both White beauty standards and the (often contradictory) standards within their cultural groups, and described these discrepancies as, “crushing” to their self-esteem [B3f]. Furthermore, women described health risks associated with attempts to obtain appearance ideals, including the use of, “flat tummy teas” that “put [a friend] in the hospital” [B3e]. Participants recommended that programs acknowledge the harms associated with these efforts to conform to beauty ideals relevant to their racial or ethnic identities.

### Harm of appearance-related comments

Women across groups repeatedly discussed the negative impact of appearance-related comments on their (and others’) body image (e.g., “I’m so fat,” [W2a, B3c, & L1b] “She’s gotten really fat” [L2a]). Participants emphasized the need for education about the consequences of these comments (e.g., body dissatisfaction), and training in how to respond (e.g., challenging negative self-talk).

### Resource limitations

Across groups, women repeatedly highlighted the need for resources challenging misconceptions about EDs in women of color. They expressed the belief EDs are, “not much of a big thing that you would see in most Latin cultures” [L1c] and “not a Black thing” [B2c]. White individuals reported believing that they are *more* prone to EDs than Black and Latina women, because resources disproportionately, “talk about EDs with White women” [W2a].

Groups also provided feedback on a DBI excerpt. Black and Latina participants reported difficulty relating to the individual described in the role-plays, “I don’t hear our race,” [B2b], and, “Is anyone Hispanic in that scenario?” [L1a]. One White woman also commented on the lack of representation, “I didn’t even realize when you were reading these things that nowhere in these like role-plays is the girl described…Yet,…I was picturing a White girl”[W2d].

Latina women further indicated that, in general, ED prevention excludes diverse women, highlighting the limited, “resources we can identify with” [L1d]. Black women similarly noted, “Most of the time, when I hear about anorexia, it’s not about Black people…” [B1b] Consequently, Black and Latina women agreed that they do not think of women within their cultural groups as susceptible to EDs. Participants expressed the importance of all women being, “aware of what can happen to them, and not just like White people, everyone” [L2c].

### Stigma/minimization of mental health

In addition to discussing recommendations for culturally adapting programs, Black and Latina women highlighted the stigmatization and minimization of mental health within their cultures. They commented that mental health concerns are, “not considered real problems” [B2c] within their cultural groups. Further, Latina women expressed reluctance to seek help for fear of generating, “extra stigmas” [L1a].

### Inclusive programming barriers

Discussion of barriers to inclusive programming highlighted: stereotypes, vulnerability, diversity, and time. Women across groups noted that stereotypes are incompatible with culturally sensitive programming, commenting, “We should not be shaming cultures,” [W1c]. Participants also expressed concern regarding what others might think about their engagement in a program addressing EDs and weight management, as it demonstrates vulnerability.

Further, women across groups highlighted distinct beauty standards as an inclusive programming challenge. “If you specify one [beauty ideal], someone might think, ‘Oh, that is not how I see it,’” [L1c]. More generally, participants highlighted challenges relating to individuals from disparate backgrounds. Participants agreed, “It’s easier to talk [in homogenous groups] because you feel like you can relate more to the people around you” [B3d]. Latina women also described lack of time as a barrier to program engagement. One commented, “I feel like in Latina culture, it is more like we’re working or studying or doing all this stuff, but there, in other cultures, it is very common that they don’t have a job and they just go to school…” [L1b].

### Inclusive programming facilitators

Participants recommended several strategies to enhance group cohesion, including, skilled facilitation, validation of individual differences, fostering connection, defining appearance broadly, and diversity. Specifically, participants noted facilitators, “have to know what we’re talking about” regarding cultural differences [L2b]. Women emphasized the importance of leaders, “do[ing] some research” [B3e] regarding multicultural concerns so, “they’re familiar with [cultural considerations]” [L2b] and able to understand participants’ lived experiences. Participants also recommended facilitators foster safety and have strong group management skills. If harmful comments are made, “The facilitator has to…take control of the conversation” [B2f]. Moreover, they noted that leaders need to ensure that participants are, “sensitive about weight,” [W1b], and, “no one fat shames or skinny shames anyone” [W1e].

Participants also considered validation and respect of individual differences crucial to group cohesion. They indicated that participants in heterogeneous groups need to be, “sensitive to” individual differences [L2b]. Women suggested acknowledgement of diverse viewpoints so, “you’re not catering to one specific group” [B1b]. Moreover, they recommended facilitators intentionally promote connection, which they viewed as achievable regardless of cultural differences. “You just have to make everyone feel like there’s something in here that they can identify with…We’re all women…We’re all in college… just point out the things that are in common and not the things that we physically can see that aren’t in common” [B2f].

Women also identified the importance of both defining appearance broadly, and promoting body acceptance; (“teaching about things involving self-worth and acceptance” [B1a] might improve everyone’s appearance satisfaction). “[Programming] should be less about how our bodies should look, …and be more like appreciating your own body” [W1b]. Indeed, participants said they would, “feel more comfortable being a part of something that promoted wellness…rather than focus on how to avoid [EDs and obesity]” [W2d].

Women across groups thought programs with racially and ethnically diverse members increase awareness of the experiences of individuals different from oneself. “If you have diversity in the group then people would realize, oh…like anybody and everybody can deal with it” [B1a]. They further noted disadvantages of homogeneous groups, recalling that interactions with similar others can yield, “a lot of the same information” [B2c]. Conversely, connecting with diverse individuals can show, “…that people are going to be different than you, but you may relate to them” [L1c].

### Manual development

Following completion of the focus groups, the (Blinded) manual was developed. Focus group data informed modifications to the four-session, clinician-lead, DBI-based Body Project manual ([55] Table 1). Attention was given to removing the emphasis on thinness, and instead, emphasizing beauty ideals generally, to represent pressures experienced by Black and Latina women more accurately. Activities/role plays were revised to highlight overall appearance pressures, and incorporate more culturally relevant examples, including, “I just started wearing a waist trainer. It’s supposed to help me get that perfect hourglass shape!” Some examples regarding the thin ideal were retained to ensure content acknowledged the continued focus on this ideal in the media. However, more diverse appearance standards were highlighted throughout, and intersections among ideals were discussed.

**Table 1:** Recommendations for ED prevention

Theme	Recommendation
Diverse beauty standards across groups	- Incorporate diverse appearance narratives - Address physical features influencing attractiveness beyond body shape and size (e.g., hair, skin tone) - Include information emphasizing appearance issues relevant to women of color (e.g., family influences, curvy ideal)
Lack of acknowledgement of racialized beauty standards and cultural appropriation	- Address racialized aspects of beauty - Retain some discourse surrounding thinness - Discuss cultural appropriation
Culturally-specific impacts of standards	- Target consequences of appearance discrepancies for women of color (e.g., microaggressions) - Reflect harmful consequences of beauty ideals pertinent to women of color - Address detrimental aspects of appearance ideals beyond thinness
Harm of appearance-related comments	- Include education regarding consequences of weight-related talk and deterrence strategies
Stigmatization of mental health	- Acknowledge stigma - Expand outreach and engagement within Black and Latina communities
Limitations of available resources	- Address misconceptions (e.g., EDs only affect White women) - Note prevalence and presentations of EDs in women of color
Facilitators and barriers to inclusive programming	- When possible, offer individuals options for either racially and ethnically homogenous or heterogenous groups - skilled facilitation - validation of individual differences - fostering connection - defining appearance broadly (emphasize body acceptance over appearance)

Modifications to the four-session Healthy Weight manual were informed by focus group data indicating a preference for positive framing and body acceptance [56]. For example, the definition of the, “healthy ideal” in Stice and colleagues’ Healthy Weight manual stated, “the healthy ideal is a reasonably slender body, but one that has muscles and fat as well. Each is natural and serves important functions.” As this explanation suggests that an ideal body exists, this definition was changed to that used in the two-session, peer-lead Body Project, which describes the healthy ideal as, “the way your unique body looks when you are doing the necessary things to appropriately maximize your physical health, mental health, and overall quality of life” [3]. Additionally, explicit nutritional and activity guidelines were removed from the Healthy Weight manual based on research suggesting that the inclusion of dietary principles weakened intervention efficacy [54]. Additional recommendations for facilitator training and supervision are reviewed in the following section.

## Discussion

Despite the high prevalence of eating concerns among women across ethnic and racial groups, the cultural relevance of prevention programming addressing these issues is not well-understood. This study investigated Black, Latina, and White college women’s perceptions regarding appearance pressures and current prevention resources, in an effort to improve the cultural relevance of two established programs. Participants noted that existing prevention resources emphasized thinness, limiting their cultural relevance. They also offered suggestions for improving prevention programming.

### Cultural adaptations

Participants across groups agreed on the need for more culturally sensitive ED and healthy weight management resources. Previous research also highlights the lack of such resources, especially for Latina and Black women [40]. Black and Latina women also noted limited awareness of EDs among women of color. Research suggests both providers and patients are more likely to recognize EDs in White individuals, compared with other racial and ethnic groups, even when symptom presentations are similar [47]. Moreover, some have asserted that because Black women endorsed lower levels of body dissatisfaction, they were less susceptible to EDs than White women [15]. However, more recent findings, and current results, suggest that Black women *do* experience body dissatisfaction; however, their body and appearance ideals often differ from those of White women [26]. Latina and Black participants emphasized the importance of including diverse appearance ideals in prevention programming. Specifically, they described how non-weight focused aspects of appearance (e.g., skin tone) significantly impacted their body image. Prior research has similarly highlighted both the negative impact of issues like colorism on body image, and the need to expand definitions of body ideals beyond thinness [35]. Overall, findings underscore the need for culturally sensitive programming.

Consistent with past research, Black and Latina participants noted that mental illness is often stigmatized or minimized within their culture [31, 39]. Results are consistent with research highlighting stigmatization of EDs within marginalized groups [11], and are concordant with research highlighting the misconception that EDs do not affect Black or Latina women [40]. Culturally sensitive prevention should acknowledge mental health stigma, and raise awareness of EDs in women of color.

### Implementation recommendations

In addition to yielding important information about content changes for these programs, participants offered suggestions for facilitators. This feedback highlights the importance of having facilitators with significant multicultural knowledge, awareness, and skills with the specific population targeted. Facilitators also need to demonstrate cultural humility, and maintain safe spaces in which participants feel comfortable sharing sensitive information. Facilitators should validate participants’ lived experiences, and have awareness of the influence of factors, such as systemic racism and cultural appropriation, on the issues discussed in group. Development of these skills is an ongoing process, but, at a minimum, facilitators require training in group and multicultural counseling. Furthermore, supervision should focus on the creation and maintenance of safe, cohesive groups that optimize cultural sensitivity. These recommendations are supported by the results of a meta-analysis of Body Project trials, which indicated that both training and supervision of facilitators, regardless of their experience level (e.g., clinicians or peers), were among the strongest predictors of outcomes [51]. Similar findings regarding the importance of supervision were reported in a recent investigation of factors affecting the effectiveness of peer-led Body Project groups [53].

Participants also had diverse views regarding the ideal group composition, with some preferring ethnically and racially homogenous groups, while others (across races) considered heterogenous groups beneficial. Stice and colleagues’ Body Project meta-analysis indicated that intervention effects were strongest when groups had a higher proportion of ethnically and racially minoritized members [51]. However, these data should be interpreted cautiously, as their analysis compared all individuals of color with European Americans. We recommend, (when resources allow), that members have the option to choose group composition type.

### Limitations

Results should be interpreted within the context of limitations. In particular, the sample consisted of undergraduates, limiting generalizability. In addition, because immigration status was not assessed, it is not known how generational status or acculturative stress might have influenced participants’ responses. Given this investigation’s focus on prevention, individuals with BMIs ≥ 30 were not included; thus it is not known if they might have had different perspectives on the HWI.

Importantly, this study was conducted to inform the development of an integrated ED and HWI program [46]. Thus, it is not a comprehensive investigation of all appearance ideals present in these groups. Despite limitations, this study provides: insight into appearance ideals and the limitations of extant resources, and suggestions for future efforts. Overall, findings highlight the heterogeneous appearance ideals of racially and ethnically diverse women. Yet, ED prevention continues to emphasize thin ideal internalization, and weight management continues to center weight loss, implying a thin ideal [58]. Enhancing programs’ cultural sensitivity could improve outcomes, particularly for Black and Latina women. Future research should also aim to adapt these interventions for other racial and ethnic groups, as culturally sensitive interventions might also benefit other groups who have historically been under researched.

## Data Availability

Data are available upon request.

## Appendix A: focus group questions

### Appearance ideals & perceived influences on development

- Most of us are aware of various appearance ideals or beauty standards that are present in our culture.
  o What are some of these ideals that stand out to you? *[Probe to investigate which ones they pay most attention to].*
  o What, if any, are the appearance ideals that you feel are “pushed” on you or that you are “expected” to strive towards, even if you don’t agree with them?
- Which appearance or beauty ideals, if any, do you personally strive to achieve?
- What are specific aspects of appearance that you consider beautiful?
  o What does your ideal of the “perfect woman” look like?
  o What do you think makes these aspects of appearance beautiful?
  o Where do you think we learn that these aspects of appearance are beautiful?
- With all the emphasis on appearance present in the media, it is hard for any woman to be completely satisfied with her body. In fact, in some circles, it's common to put down your appearance, and say or think negative things about your weight or shape.
  o How do media messages impact the way you feel about your body?
  o What parts of your appearance are you dissatisfied with, and why?
  o What parts are you satisfied with, and why?
- In today’s society, there is a lot of emphasis on appearance. How much would you say that your appearance influences your evaluation of yourself?
  o Where have you received the message that your appearance influences your self-worth? (e.g., media, parents, friends)
  o How do these messages from outsides sources influence your view of your self?
- Who, if anyone, do you compare yourself to in terms of appearance? (e.g., peers, family members, actors, models)
  o How do your peers or close friends impact the way you feel about your body?
- How does the racial/ethnic identity of your peers influence the way you compare yourself to them?
  o What specific aspects of your appearance do you compare to others?
  o How do these comparisons make you feel about your self?
- How have the appearance ideals you’ve mentioned influenced the way you live your life?
  o Do you do anything differently in your day-to-day life because of these appearance ideals?
  o Can you think of any ways that your (or society’s) appearance ideals have negatively impacted you?
  o Positively impacted you?
- What types of statements do you hear women commonly make when they are “down talking,” or talking badly about, their appearance?
  o What types of statements do you hear women commonly make when they are body shaming, or appearance shaming, other women?
- Do the type of comments you hear differ based on racial/ethnic identity of the women involved?
- What would be different about your life if you were satisfied with the way you looked?
  o What would be different about your life if you could wave a magic wand and the parts of your appearance that you were dissatisfied with were changed?
- Do you know people who are satisfied with their appearance?
  o What is different about how they think about themselves?
- Do you have any ideas for ways to feel better about how you look?

### Differences in appearance ideals among different ethnic & racial groups

- How do you think your racial or ethnic background affects your appearance ideals?
  o *[Probe to see if they feel their racial or ethnic background pressures them to look, or try to look, a certain way].*
- How does your racial or ethnic background affect how you feel about your appearance?
- What types of beauty ideals were seen as important in your family and/or culture?
  o What do your family and/or culture tell you will happen if you achieve these beauty ideals?
- What if you do not achieve these beauty ideals?
  o What types of beauty ideals were seen as unimportant in your family and/or culture?
- How are your personal beauty standards different from people in your same racial/ethnic group?
  o How are they the same?
- How do your personal beauty standards compare to the beauty standards of people in other racial/ethnic groups?
  o How are they different?
  o How are they the same?

### Rationale for proposed intervention

- As you probably know, both obesity and eating disorders are common and serious issues that disproportionally affect young adult women. Programs to prevent the development of obesity and eating disorders exist, yet they have been largely implemented in White populations. The most effective eating disorder prevention program currently available focuses on the Western (White) thin ideal as the target for change. Yet, in a previous study, many women at VCU told us that this ideal wasn't relevant for them. Therefore, the goal of this focus group is to gather information about how to design a prevention program that is more culturally sensitive and in order to combat eating disorders and obesity in a broader range of college women.
- What do you think about the idea of developing a prevention program that targets obesity and eating disorders at the same time?
  o Probe to see if they feel this type of prevention program is needed.
- Do you feel pressure to achieve the White thin ideal?
  o Do you see the thin ideal affecting people in your same racial/ethnic group? In what ways?
  o Do you think the thin ideal affects women of different racial/ethnic groups in different ways? If yes, probe for these differences and how they might manifest in across racial/ethnic groups.
- •What do you think is the greatest risk factor (in terms of appearance) for an eating disorder and/or obesity?
  o What about for people in your same racial/ethnic group?
- I mentioned earlier that the most effective eating disorder prevention program to date focuses on the White, European thin ideal. As part of this program, participants engage in role-plays in which they try to discourage other people from pursuing the thin ideal. I’m going to review some of these existing role-plays. I would like you to provide feedback for how the role-plays can be improved to benefit women like you. [Probe for ways to improve that are related to culture, but also see if there are other ways they mention role plays could be changed to enhance relevance.]
  o In these role-plays, the leaders play a person who is obsessed with the thin ideal. The participant’s job is to convince the leaders to not pursue the thin ideal. There are three different characters that the leader can play. I’ll read the script for each character, and ask for your critiques and feedback.
    • Character 1: I am going to play a friend who is obsessed about how my body will look for spring break. I’m dying to have a flat stomach, so I have put myself on a vegetarian diet because meat contains an outrageous amount of fat, which will make me huge and disgusting. In order to lose as much weight as possible, I also refuse to eat carbohydrates. I did this last year to lose weight for spring break but started too late to get the effects I wanted. So this time, I started 5 months ago. I’m dieting because I know I will have to wear a bikini on the beach. Whenever my friends and I mention spring break all I can think about is how I can’t wear a swimsuit in front of everyone if I don’t have an amazingly flat stomach.
      ▪ Probe for critiques of role-play & feedback on role play especially (but not exclusively) ways to make it more culturally relevant to the current focus group participants
    • Character 2: I am going to play a freshman who is trying to get into a sorority. I’m very concerned about gaining the freshman fifteen because I know if I do, no one will want to be my friend or give me a bid. I weigh myself at least four times every day to make sure that I’m losing weight, or a least not gaining any. If my weight is higher than it was the last time, I skip my next meal and hope for better results at the next weight in. Sometimes I’m late for class because I have to get back to my dorm room between classes to weigh myself or I won’t be able to focus on anything else. If I don’t start losing weight faster, then I will start skipping two meals every time my weight doesn’t go down by at least ¼ of a pound.
      ▪ Probe for critiques of role-play & feedback on role play especially (but not exclusively) ways to make it more culturally relevant to the current focus group participants
    • Character 3: I am going to play a friend who is exercising three times a day because I am trying to get a thigh gap to make me more attractive to the person I’m dating. I run 3 miles after breakfast, lunch, and dinner every day because if I don’t, I feel super gross. It’s like I can feel the food in my stomach moving straight to my thighs and accumulating there. I run even if I’m sick or injured because I know I will get fat thighs if I skip even one work out. The person I’m dating says they won’t date girls with fat legs and in the past they have teased me for gaining weight. I stopped losing weight last week, so I think I need to amp up the mileage.
      ▪ Probe for critiques of role-play & feedback on role play especially (but not exclusively) ways to make it more culturally relevant to the current focus group participants

### Cultural acceptability of proposed intervention

- How could researchers design an intervention to prevent eating disorders and obesity in a culturally diverse group of young women?
- What types if things could help improve the cultural acceptability of the intervention?
- What types of things might hinder the cultural acceptability of the intervention?
- How can we best design an intervention to address multiple aspects of appearance and remain respectful of racial/ethnic differences?

### Barriers & Facilitators to programming for obesity & EDs

- Would you be interested in participating in the intervention that we have talked about to learn ways to accept your appearance, eat better, and live a healthier life? Why or why not?
- What types of things should be included in this type of intervention?
- What would make you more likely to participate in the intervention?
  o What would make you less likely?
- What length of time do you think is best (i.e., how many weeks) for this type of intervention?
  o What times of the year?
  o Times/days of the week?
  o Lengths of sessions?
- What would make it most challenging for you to be in this type of intervention?
- What would make you likely to stay in such an intervention?

### Strategies for creating cohesive intervention cohorts

- We know that discussing personal experiences surrounding food and weight can be uncomfortable, and that these discussions can be even more uncomfortable if you feel different than or judged by other group members. We are hoping to minimize discomfort and create intervention groups that feel safe and encourage connection.
- Would you feel comfortable in this type of intervention group?
  o What would make you feel more comfortable?
  o What would make you feel less comfortable?
- Many people prefer being in groups with people similar to themselves. Might the cultural backgrounds of others in the group (i.e., race/ethnicity, religion, socio-economic status) influence how comfortable you would feel? *Probe for each background factor specifically and make sure they are all addressed.*
  o What about other factors like body size, age, year in school? *Again probe for a response to each factor.*
  o Anything else about other group members that might be important to you?
- What about the cultural background of the intervention leader? Are there any features of the intervention leader that would influence how comfortable you would feel (i.e., race/ethnicity, religion, socio-economic status, body size, age, year in school)?
- How do you feel about participating in a more homogenous group?
  o What are the pros and cons of a homogenous group?
- How do you feel about participating in a more diverse group?
  o What are the pros and cons of a diverse group?
- If a homogenous group is not feasible, what things can we do to create a cohesive group?
- How can we facilitate a safe and open group environment among women of various cultural backgrounds?
